# Evaluating the effect of care around labor and delivery practices on early neonatal mortality in the Global Network’s Maternal and Newborn Health Registry

**DOI:** 10.1186/s12978-020-01010-w

**Published:** 2020-11-30

**Authors:** Archana B. Patel, Elizabeth M. Simmons, Sowmya R. Rao, Janet Moore, Tracy L. Nolen, Robert L. Goldenberg, Shivaprasad S. Goudar, Manjunath S. Somannavar, Fabian Esamai, Paul Nyongesa, Ana L. Garces, Elwyn Chomba, Musaku Mwenechanya, Sarah Saleem, Farnaz Naqvi, Melissa Bauserman, Sherri Bucher, Nancy F. Krebs, Richard J. Derman, Waldemar A. Carlo, Marion Elizabeth M. Koso-ThomasMcClure, Patricia L. Hibberd

**Affiliations:** 1 Lata Medical Research Foundation, Nagpur, India; 2 Datta Meghe Institute of Medical Sciences, Wardha, India; 3 School of Public Health, Boston University, Boston, MA USA; 4 RTI International, Durham, NC USA; 5 Department of Obstetrics and Gynecology, Columbia University School of Medicine, New York, NY USA; 6 KLE Academy Higher Education and Research, J N Medical College, Belagavi, Karnataka India; 7 Moi University School of Medicine, Eldoret, Kenya; 8 Instituto de Nutrición de Centroamérica y Panamá, Guatemala City, Guatemala; 9 University Teaching Hospital, Lusaka, Zambia; 10 Aga Khan University, Karachi, Pakistan; 11 University of North Carolina at Chapel Hill, Chapel Hill, NC USA; 12 University of Indiana, Indianapolis, IN USA; 13 University of Colorado School of Medicine, Denver, CO USA; 14 Thomas Jefferson University, Philadelphia, USA; 15 University of Alabama at Birmingham, Birmingham, AL USA; 16 Eunice Kennedy Shriver National Institute of Child Health and Human Development, Bethesda, MD USA

**Keywords:** Neonatal mortality, Early neonatal mortality, Quality of care, Labor and delivery care, Newborn care, Composite index, Intrapartum care, Postpartum care, Early neonatal period, Low income countries, Lower middle-income countries, Essential newborn care, Global network

## Abstract

**Background:**

Neonatal deaths in first 28-days of life represent 47% of all deaths under the age of five years globally and are a focus of the United Nation’s (UN’s) Sustainable Development Goals. Pregnant women are delivering in facilities but that does not indicate quality of care during delivery and the postpartum period. The World Health Organization’s Essential Newborn Care (ENC) package reduces neonatal mortality, but lacks a simple and valid composite index that measures its effectiveness.

**Methods:**

Data on 5 intra-partum and 3 post-partum practices (indicators) recommended as part of ENC, routinely collected in NICHD’s Global Network’s (GN) Maternal Newborn Health Registry (MNHR) between 2010 and 2013, were included. We evaluated if all 8 practices (Care around Delivery – CAD), combined as an index was associated with reduced early neonatal mortality rates (days 0–6 of life).

**Results:**

A total of 150,848 live births were included in the analysis. The individual indicators varied across sites. All components were present in 19.9% births (range 0.4 to 31% across sites). Present indicators (8 components) were associated with reduced early neonatal mortality [adjusted RR (95% CI):0.81 (0.77, 0.85); p < 0.0001]. Despite an overall association between CAD and early neonatal mortality (RR < 1.0 for all early mortality): delivery by skilled birth attendant; presence of fetal heart and delayed bathing were associated with increased early neonatal mortality.

**Conclusions:**

Present indicators (8 practices) of CAD were associated with a 19% reduction in the risk of neonatal death in the diverse health facilities where delivery occurred within the GN MNHR. These indicators could be monitored to identify facilities that need to improve compliance with ENC practices to reduce preventable neonatal deaths. Three of the 8 indicators were associated with increased neonatal mortality, due to baby being sick at birth. Although promising, this composite index needs refinement before use to monitor facility-based quality of care in association with early neonatal mortality.

*Trial registration* The identifier of the Maternal Newborn Health Registry at ClinicalTrials.gov is NCT01073475.

## Background

By the end of 2015, global childhood mortality and the maternal mortality ratio (United Nations’ [UN] Millennium Development Goals 4 and 5 respectively) had improved globally, but neither goal target was reached [1]. An estimated 2.5 million neonatal deaths occurred in 2018 accounting for 47% of deaths in children under age 5 [2–4]. The focus on reducing maternal, childhood and particularly neonatal mortality continues to be a part of the UN’s Sustainable Development Goal 3 with new targets for 2030 [5].

High quality of care during pregnancy, labor and delivery, and immediately post-partum is critical to reducing maternal, perinatal and neonatal mortality [1, 6, 7]. This need is being partly addressed by an increase in access to institutional deliveries and presence of a skilled birth attendant at delivery, but access to health care providers does not guarantee that recommended interventions will be provided. The World Health Organization (WHO) has recently developed a framework and standards for health care facilities that includes 8 overarching standards and 352 quality measures [8]. However, it is challenging to assess quality of maternal and newborn care based on these standards and criteria as noted by Brizuela et al. [9]. Guidance is needed to address priority measures.

There is a current focus on developing simple and valid indicators of facility-based quality of care at the time of birth to enable rapid assessment of quality and institute data-driven action to improve outcomes. Recently published tools such as the WHO’s Safe Childbirth Checklist address this void [10–13] but require significant data collection efforts. However, in a large trial in Utter Pradesh, India, use of the Safe Childbirth Checklist program did not result in reduced maternal or perinatal mortality [14], while a quasi-experimental study of the checklist tool resulted in an 11% reduction in stillbirths and very early neonatal deaths within 3 days of birth [15].

Prior to the Safe Childbirth Checklist studies, our group had focused on just eight Care Around Delivery (CAD) indicators derived from Essential Newborn Care (ENC) practices and recommended by WHO [16–19]. All 8 indicators were routinely collected in NICHD’s Global Network’s (GN) Maternal Newborn Health Registry (MNHR) between 2010 and 2013. Five of the indicators were intra-partum and are also known as the 5 “cleans” to reduce the risk of neonatal sepsis. These include clean hands, clean cord tie, clean cord, clean surface and clean blade. The 5 cleans are usually addressed by providing clean delivery kits. Three of the immediate post-partum indicators included early initiation of breast feeding within 1 h of birth, skin to skin practices immediately after birth and bathing delayed until 6 h after birth. Since presence of ENC and immediate neonatal care practices are associated with reduced early neonatal mortality [20, 21], we evaluated whether occurrence of all of these 8 indicators of ENC that were available in the MNHR would also be associated with reduced early neonatal mortality. Our hypothesis was that occurrence of all 8 CAD indicators (composite index) was associated with early neonatal mortality (days 0–6 of life). We also explored the effects of the individual indicators on very early mortality.

## Methods

### Study design, setting and participants

The MNHR, a study conducted by the *Eunice Kennedy Shriver* National Institute of Child Health and Human Development’s (NICHD’s) Global Network, is a multi-site research network representing partnerships of U.S. and international investigators that from 2010–2013 were at study sites in Argentina, Guatemala, India (2 sites: Nagpur and Belgaum), Pakistan, Kenya, and Zambia. Argentina was excluded from this analysis because the level of obstetric care in Argentina was much higher than in the other sites. Data from the MNHR from 2010–2013 were the only years where all 8 indicators of care around delivery were collected. Since its start in mid-2008, MNHR has registered approximately 70,000 pregnant women and their babies annually in rural and semi-urban communities in the countries listed above. Detailed methods utilized by the MNHR have been previously published [22]. The MNHR registry collects data on outcomes of trials hence it has a clinical trial registry number. Briefly, pregnant women in the catchment area of 6–24 geographic clusters in each country are enrolled into the registry as early in their pregnancy as possible. The enrollment target in all participating communities is at least 95% of pregnant women. Outcomes for these women are tracked throughout their pregnancy, delivery and during the post-partum period with follow-up data collected within 7 days of delivery and around 42 days post-partum.

#### Ethical clearance

The Institutional Review Boards and Ethics Research Committees of all participating institutions, and the appropriate Ministries of Health of the respective countries approved the MNHR. Prior to initiation of the study, approval was obtained from the participating communities through sensitization meetings. Individual informed consent for study participation is required from each study participant. No monetary reimbursements are provided to study participants nor to the communities participating in the study. A Data Monitoring Committee, appointed by NICHD, oversees and reviews the study at annual meetings.

### Data collection tools, procedures and quality control

Data on the enrolled women are collected by trained health workers at 3 time points: at enrolment (as early as possible in pregnancy: age, height, weight, parity and educational status), at delivery (within one week of birth: date of delivery, birth weight, mode of delivery, neonatal status, place of delivery), and at 42 days post-partum (maternal mortality, neonatal survival, and hospitalizations of the mother or baby). Senior Foreign Investigators (on-site primary investigators) at all sites are trained centrally. They then train their sites’ data collectors prior to collecting study data. Data collected on paper are entered into a database at a site-based data management center and transmitted to a central data coordinating center at Research Triangle Institute International (RTI), Durham, NC, USA. RTI monitors the data with monthly reports of data quality (completeness and timeliness) and edit reports to identify out of range or inconsistent data that are then addressed by the site staff as well site visits.

#### Eligibility criteria

Pregnant women included in this analysis were screened, consented and enrolled in the MNHR between January 2010 and December 2013 during which time 2 versions of the study form were used for data collection. Because of varying site implementation, several sites had modified study dates (Guatemala’s study period was March 2010 to December 2013; Belagavi and Pakistan’s study periods were January 2010 to November 2013).

Pregnant women were excluded in the pre- and intra-partum period if:

i ENC and post-partum care were not relevant (maternal death prior to labor and delivery, miscarriage, medically terminated pregnancy, stillbirth or home delivery);
j the pregnancy was a multiple gestation (to avoid double counting pregnant women).

Pregnant women were also excluded in the post-partum period if:

i there was no study outcome (neonatal vital status at day 42 post-partum);
j any of the 8 care-around- delivery (CAD) indicators were missing.

#### Outcomes

The primary outcome was early neonatal mortality (per 1,000 live births) on days 0–6 of life. We also explored secondary outcomes of very early neonatal mortality (per 1,000 live births) on days 0–1 of life and late early neonatal mortality on days 2–6 of life.

#### Exposure

Our exposures of interest were presence of all 8 dichotomous CAD indicators, the first 5 relating to intra-partum of care indicators from ENC and the last 3 relating to recommended immediate post-partum care of the baby:

i CAD1: Delivery in a hospital versus not in a hospital
j CAD2: Skilled birth attendant at delivery—present versus absent
k CAD3: Fetal heart rate assessed prior to delivery—assessed versus not assessed
l CAD4: New gloves for delivery—used versus not used
m CAD5: Clean cord practices—clean razor used versus not used
n CAD6: Early initiation of breast feeding (within 1 h of birth)—done versus not done
o CAD7: Skin-to-skin practices (immediately after birth)—provided versus not provided
p CAD8: Delayed bathing (> 6 h of birth)—done versus not done

For the purposes of this analysis, a hospital was defined as a health facility that provides inpatient services for 24-hours/day, medical and nursing care for medical and surgical diagnosis, treatment and rehabilitation, is staffed by at least one physician and may also provide outpatient services. A clinic was defined as providing facilities for labor and delivery (vaginal only). Facility deliveries encompass deliveries that occurred in both clinics and hospitals.

We also created a composite index categorizing all births into one of three categories: (i) all 8 intra-partum and post-partum CAD indicators; (ii) all 5 intra-partum indicators and 0–2 post-partum indicators; (iii) all other combinations of CAD.

#### Covariates

Covariates included parity, delivery mode, gestational age < 34 weeks (moderate or early preterm) [23], birth weight < 1500 g, presence of maternal condition (any of obstructed/prolonged labor/failure to progress, major ante-partum hemorrhage, major post-partum hemorrhage, hypertensive disease/severe pre-eclampsia/eclampsia or breech/transverse or oblique lie) and presence of neonatal condition (any of congenital anomaly, breathing problems, feeding problems, high fever, hypothermia, convulsions or having been resuscitated).

### Statistical analysis

Analyses were conducted on 150,848 deliveries with data on all eight CAD indicators. We computed summary statistics (for example, n and proportions) for characteristics of mother and child, exposures (CADs and composite index) and outcomes (early neonatal mortality: days 0–6, 0–1, and 2–6) for each of the 7 sites. Log-binomial and cumulative logit models using generalized estimating equations to account for the correlation of outcomes within cluster were used to assess whether the characteristics, exposures and outcomes varied across the sites. A similar model was used to assess the association between the covariates and the CAD-8 indicator for delayed bathing. Relative risks (RR) and 95% confidence intervals (CI) for each early neonatal mortality outcome were obtained from separate multivariable Poisson Generalized Linear Models, using generalized estimating equations to account for the correlation of outcomes within cluster, including exposures and site with and without adjustment for other covariates. Models were run separately for each of the 8 CAD variables as well as the presence of all 8 CAD indicators. All analyses were conducted in SAS 9.4 (SAS Inc., Durham, NC). A two-sided p-value < 0.05 was considered to be statistically significant.

## Results

**Fig. 1:**
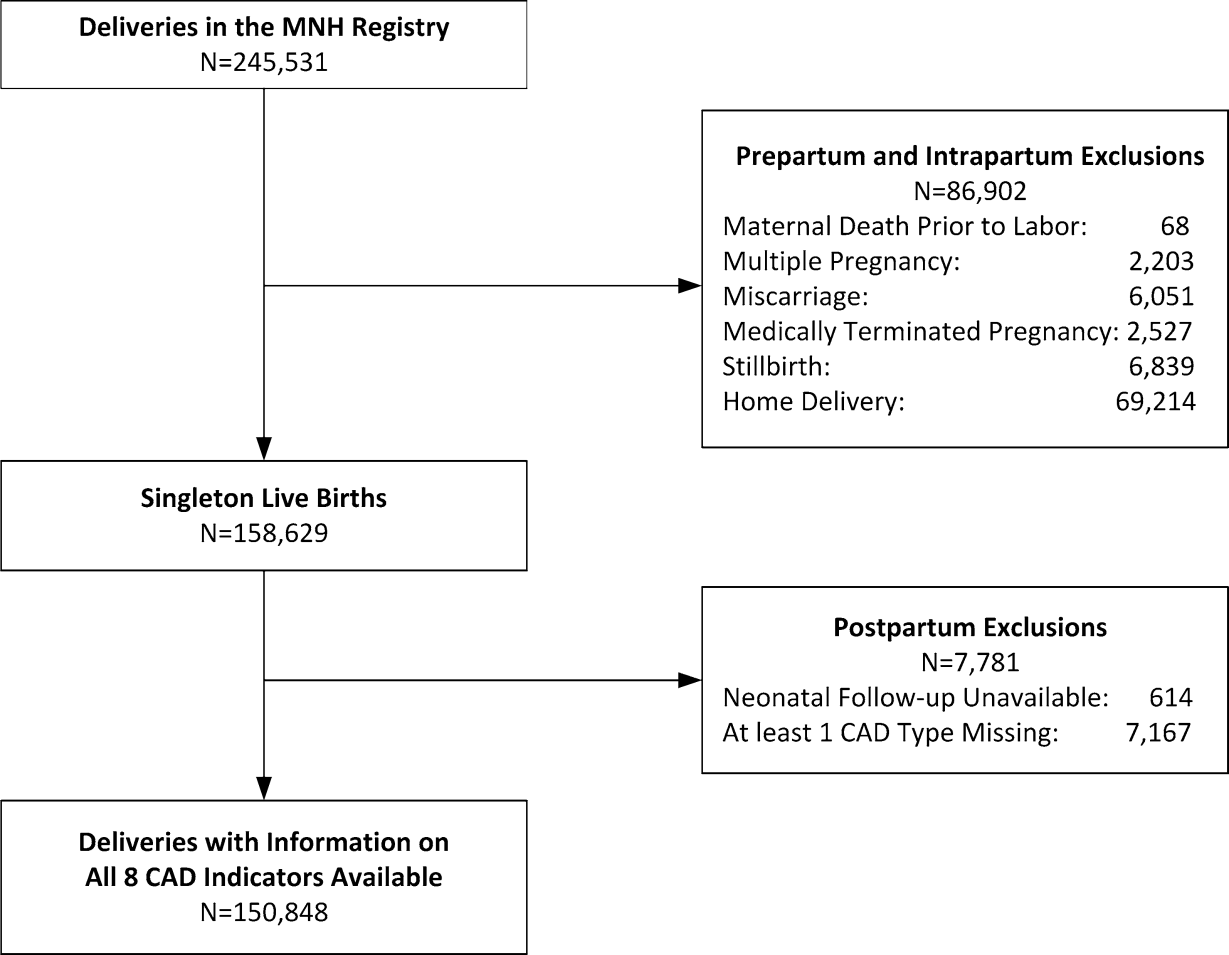
Flow diagram

A flow diagram describing the study population is displayed in Fig. 1. Of the 245,531 deliveries in the MNH Registry between January 2010 and December 2013, the following were excluded: 86,902 during the pre-partum and intra-partum periods and 7,781 in the post-partum period, for reasons described in the methods. Results from the final sample of 150,848 singleton, live births with information on all 8 CAD indicators are reported below.

Maternal characteristics, maternal and neonatal conditions and early neonatal mortality rates for women delivering in facilities are displayed by site in Table 1. The proportion of nulliparous women varied from 27% in the Pakistan site to 49% in the Nagpur site. The percentage of Cesarean sections ranged from 1.4% of deliveries in the Zambia site to 39% of deliveries in the Guatemala site. At least one maternal condition was present in 8% of the pregnant women in the Zambia site to 43% in the Pakistan site, while at least one neonatal condition was present in 4% of live born neonates in the Zambia site to 25% in the Pakistan site. The proportion of all facility births with a gestational age < 34 weeks was lowest in the Guatemala (2%) site and highest in the Zambia site (7%), while the percentage of very low birth babies born alive was < 1% across all sites. Early neonatal mortality rates (per 1,000 live births) on days 0–6 varied from 11.7 in the Kenya site to 42.3 in the Pakistan site.

**Table 1:** Distribution of maternal characteristics, maternal conditions and neonatal conditions by site

	Total	Zambia	Kenya	Guatemala	Belagavi	Nagpur	Pakistan	p-Value^c^
Live births, N	150,848	16,069	13,989	6351	55,909	35,458	23,072	
Parity, N (%)	150,138	16,053	13,772	6350	55,503	35,451	23,009	< .0001
0	59,938 (39.9)	5052 (31.5)	4623 (33.6)	2496 (39.3)	24468 (44.1)	17202 (48.5)	6097 (26.5)	
1–2	66,095 (44.0)	5972 (37.2)	5222 (37.9)	2268 (35.7)	27,771 (50.0)	17,389 (49.1)	7473 (32.5)	
3+	24,105 (16.1)	5029 (31.3)	3927 (28.5)	1,586 (25.0)	3264 (5.9)	860 (2.4)	9439 (41.0)	
Cesarean delivery, N (%)	150,848	16,069	13,989	6351	55,909	35,458	23,072	< .0001
Yes	22,810 (15.1)	233 (1.4)	365 (2.6)	2,484 (39.1)	8,183 (14.6)	7,297 (20.6)	4,248 (18.4)	
No	128,038 (84.9)	15,836 (98.6)	13,624 (97.4)	3,867 (60.9)	47,726 (85.4)	28,161 (79.4)	18,824 (81.6)	
At least one maternal condition^a^, N (%)	150,783	16,060	13,989	6349	55,869	35,445	23,071	< .0001
Yes	29,146 (19.3)	1,209 (7.5)	3251 (23.2)	1717 (27.0)	7528 (13.5)	5423 (15.3)	10,018 (43.4)	
No	121,637 (80.7)	14,851 (92.5)	10,738 (76.8)	4632 (73.0)	48,341 (86.5)	30,022 (84.7)	13,053 (56.6)	
Gestational age < 34 weeks, N (%)	143,669	15,518	12,830	6015	53,381	34,492	21,433	< .0001
Yes	5,345 (3.7)	1,039 (6.7)	384 (3.0)	115 (1.9)	1,551 (2.9)	987 (2.9)	1,269 (5.9)	
No	138,324 (96.3)	14,479 (93.3)	12,446 (97.0)	5,900 (98.1)	51,830 (97.1)	33,505 (97.1)	20,164 (94.1)	
Birth weight < 1500 g, N (%)	150,827	16,069	13,987	6349	55,908	35,451	23,063	< .0001
Yes	840 (0.6)	51 (0.3)	23 (0.2)	35 (0.6)	295 (0.5)	220 (0.6)	216 (0.9)	
No	149,987 (99.4)	16,018 (99.7)	13,964 (99.8)	6314 (99.4)	55,613 (99.5)	35,231 (99.4)	22,847 (99.1)	
At least one neonatal condition^b^, N (%)	150,265	15,541	13,986	6332	55,892	35,448	23,066	< .0001
Yes	14,005 (9.3)	671 (4.3)	1,012 (7.2)	474 (7.5)	4,041 (7.2)	2,015 (5.7)	5,792 (25.1)	
No	136,260 (90.7)	14,870 (95.7)	12,974 (92.8)	5,858 (92.5)	51,851 (92.8)	33,433 (94.3)	17,274 (74.9)	
Early neonatal mortality, rate/1000 (95% CI)								
Days 0–6	19.4 (18.7, 20.1)	12.7 (11.0, 14.4)	11.7 (9.9, 13.4)	16.1 (13.0, 19.2)	16.9 (15.9, 18.0)	15.1 (13.8, 16.4)	42.3 (39.7, 44.9)	
Days 0–1	12.2 (11.7, 12.8)	8.0 (6.6, 9.4)	8.7 (7.2, 10.3)	6.8 (4.8, 8.8)	10.7 (9.9, 11.6)	8.0 (7.1, 8.9)	29.0 (26.8, 31.1)	
Days 2–6	7.3 (6.8, 7.7)	4.7 (3.6, 5.8)	3.0 (2.1, 3.9)	9.4 (7.0, 11.7)	6.3 (5.6, 6.9)	7.2 (6.3, 8.0)	13.7 (12.2, 15.2)	

^a^At least one of the following maternal conditions: obstructed/prolonged labor/failure to progress, major antepartum hemorrhage, major postpartum hemorrhage, evidence of hypertensive disease/severe pre-eclampsia/eclampsia or breech/transverse or oblique lie

^b^At least one of the following neonatal conditions: congenital anomaly, breathing problems, feeding problems, high fever, hypothermia, convulsions or resuscitated

^c^Wald p-values obtained from log-binomial models using generalized estimating equations to account for the correlation of outcomes within cluster

### Relationship of individual CADs to early neonatal mortality (0–6 days)

The distribution of each of the 8 CAD indicators by site are displayed in Table 2. Rates of each CAD across all sites ranged from 50% (physician attendant) to 99.7% (use of new gloves). Among facility births, the Guatemala site had > 90% deliveries by physicians and in hospitals while the Zambia and Kenya sites had only 3% of deliveries by physicians and < 30% in hospitals. The Nagpur and Belagavi sites, respectively, had 62% and 63% of the deliveries done by physicians and 70% and 73% of deliveries occurred in hospitals.

**Table 2:** Distribution of care around delivery (CAD) indicators by site

	Total	Zambia	Kenya	Guatemala	Belagavi	Nagpur	Pakistan	p-Value^a^
Live births, N	150,848	16,069	13,989	6351	55,909	35,458	23,072	
CAD1: Delivery in a hospital versus clinic, N (%)	91,437 (60.6)	3,178 (19.8)	4,162 (29.8)	5884 (92.6)	40,521 (72.5)	24,920 (70.3)	12,772 (55.4)	< .0001
CAD2: Skilled birth attendant at delivery, N (%)	75,359 (50.0)	500 (3.1)	483 (3.5)	6058 (95.4)	35,192 (62.9)	21,870 (61.7)	11,256 (48.8)	< .0001
CAD3: Fetal heart rate assessed prior to delivery, N (%)	142,177 (94.3)	15,568 (96.9)	13,340 (95.4)	6262 (98.6)	55,602 (99.5)	35,433 (99.9)	15,972 (69.2)	< .0001
CAD4: New gloves for delivery, N (%)	150,463 (99.7)	16,045 (99.9)	13,975 (99.9)	6349 (100.0)	55,882 (100.0)	35,357 (99.7)	22,855 (99.1)	< .0001
CAD5: Clean cord practices, N (%)	148,877 (98.7)	16,042 (99.8)	13,954 (99.7)	4673 (73.6)	55,789 (99.8)	35,443 (100.0)	22,976 (99.6)	< .0001
CAD6: Early initiation of breast feeding (within 1 h of birth), N (%)	113,352 (75.1)	14,685 (91.4)	12,115 (86.6)	4402 (69.3)	47,555 (85.1)	30,503 (86.0)	4,092 (17.7)	< .0001
CAD7: Skin-to-skin practices (immediately after birth), N (%)	96,602 (64.0)	14,120 (87.9)	11,369 (81.3)	3450 (54.3)	40,624 (72.7)	25,637 (72.3)	1,402 (6.1)	< .0001
CAD8: Delayed bathing (> 6 h of birth), N (%)	135,742 (90.0)	13,800 (85.9)	8,877 (63.5)	3828 (60.3)	54,053 (96.7)	35,158 (99.2)	20,026 (86.8)	< .0001

^a^Wald p-values obtained from log-binomial models using generalized estimating equations to account for the correlation of outcomes within cluster

Results from the multivariable analyses presented in Table 3 show that lower rates of early neonatal mortality in days 0–6 were associated with delivery in a hospital compared to a clinic [adjusted RR (95% CI) 0.94 (0.89, 1.00); p = 0.04], early initiation of breastfeeding [0.43 (0.39, 0.49); p < 0.0001] and skin-to-skin practices [0.79 (0.73, 0.87); p < 0.0001]. Higher rates of early neonatal mortality in days 0–6 was associated with delayed bathing (> 6 h) [adjusted RR (95% CI):1.47 (1.32–1.64); p < 0.0001], delivery attendant is a physician [1.10 (1.04–1.16); p = 0.0013)], and fetal heart rate assessed prior to delivery [1.14 (1.04–1.24); p = 0.0048]. On further analysis of our data, babies had delayed bathing when there was either a maternal complication or a neonatal condition present, and in babies with gestational age of < 34 weeks and birth weight less than 1500 g (p < 0.0001 for association of these factors with delayed bathing). The rates of neonatal mortality were significantly lower when all 8 practices (intra-partum and post-partum) occurred when compared to not having all 8 practices [adjusted RR (95% CI):0.81 (0.77, 0.85); p < 0.0001]. Results for associations of each CAD with neonatal mortality rates on days 0–1 and 2–6 were similar to the overall results (Addiitonal file 1:Tables S1 and S2).

**Table 3:** Early neonatal mortality by received care around delivery indicator

Care around delivery (CAD) indicators	Neonatal mortality 0–6 days^a^		Risk of neonatal mortality 0–6 days			
	Received care around delivery		Care versus no care, unadjusted^b^ N = 150,848		Care versus no care, adjusted^c^ N = 142,469	
	Yes n/N (rate/1000)	No n/N (rate/1000)	RR (95% CI)	P-value	RR (95% CI)	P-value
All 8 CAD indicators	167/30,006 (5.6)	2,758/120,842 (22.8)	0.26 (0.18, 0.37)	< .0001	0.81 (0.77, 0.85)	< .0001
CAD1: Delivery in a hospital versus clinic	1,901/91,437 (20.8)	1,024/59,411 (17.2)	0.87 (0.76, 1.00)	0.06	0.94 (0.89, 1.00)	0.04
CAD2: Skilled birth attendant at delivery	1,667/75,359 (22.1)	1,258/75,489 (16.7)	0.92 (0.74, 1.14)	0.44	1.10 (1.04, 1.16)	< 0.01
CAD3: Fetal heart rate assessed prior to delivery	2,585/142,177 (18.2)	340/8,671 (39.2)	0.97 (0.79, 1.21)	0.81	1.14 (1.04, 1.24)	< 0.01
CAD4: New gloves for delivery	2,905/150,463 (19.3)	20/385 (51.9)	0.72 (0.39, 1.33)	0.30	0.83 (0.60, 1.15)	0.27
CAD5: Clean cord practices	2,881/148,877 (19.4)	44/1,971 (22.3)	0.62 (0.41, 0.95)	0.03	0.97 (0.81, 1.17)	0.76
CAD6: Early initiation of breast feeding (within 1 h of birth)	691/113,352 (6.1)	2,234/37,496 (59.6)	0.09 (0.07, 0.12)	< .0001	0.43 (0.39, 0.49)	< .0001
CAD7: Skin-to-skin practices (immediately after birth)	949/96,602 (9.8)	1,976/54,246 (36.4)	0.67 (0.53, 0.86)	< 0.01	0.79 (0.73, 0.87)	< .0001
CAD8: Delayed bathing (> 6 h of birth)	2,823/135,742 (20.8)	102/15,106 (6.8)	3.10 (2.26, 4.27)	< .0001	1.47 (1.32, 1.64)	< .0001

^a^Columns present n = the number of neonatal deaths on days 0–6; N = number of live births when CAD is present or absent; and the day 0–6 neonatal mortality rate per 1,000 births within each care around delivery indicator for care present or absent

^b^Unadjusted relative risks, 95% confidence intervals and p-values are obtained from a Poisson model for early neonatal mortality including the 8 care around delivery indicators and site with generalized estimating equations to account for the correlation of outcomes within cluster. The relative risks and p-values for all 8 CAD indicators index come from a separate Poisson model including the all 8 CAD indicators index and site

^c^Adjusted relative risks, 95% confidence intervals and p-values are obtained from a Poisson model for early neonatal mortality including the 8 care around delivery indicators and site adjusting for parity (0, 1–2, 3+ (ref)), delivery mode(vaginal, cesarean (ref)), at least one maternal condition (yes, no (ref)), gestational age < 34 weeks (yes, no (ref)), birth weight < 1500 g (yes, no (ref)) and at least one neonatal condition (yes, no (ref)) with generalized estimating equations to account for the correlation of outcomes within cluster. The relative risks and p-values for all 8 CAD indicators index come from a separate Poisson model including the all 8 CAD indicators index, site and the covariates described above

### Relationship of the composite index to early neonatal mortality (0–6 days)

**Fig. 2:**
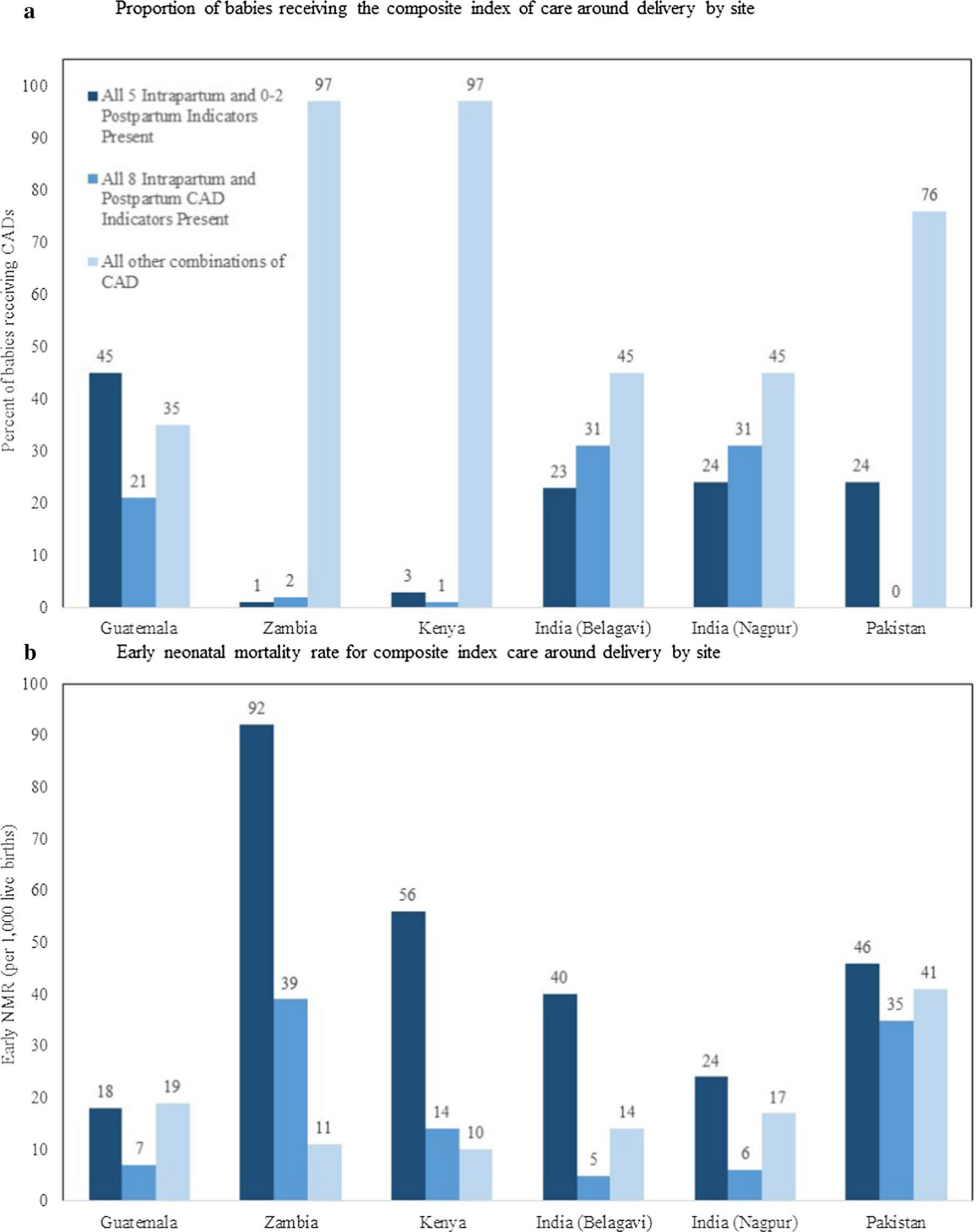
Proportion of mother/baby dyads receiving and early neonatal mortality for the composite index types of care around delivery by site

The distribution of live births in facilities that had (i) all 8 intra-partum and post-partum CAD indicators; (ii) all 5 intra-partum indicators and 0–2 post-partum indicators; (iii) all other combinations of CAD by site are presented in Fig. 2a.

All 8 CAD indicators occurred in 20% of deliveries in all sites. The pattern of early neonatal mortality rates (day 0–6) by the same categories of CAD indicators by site are shown in Fig. 2b. In all sites, mortality rates were lower when all 8 CAD indicators versus all 5 intra-partum and 0–2 post-partum indicators occurred. Mortality rates when other combinations of indicators were present varied across sites, likely associated with the heterogeneity of indicators.

### Relationship of individual CADs to the secondary outcomes (neonatal mortality in 0–1, and 2–6 days)

The relationships were similar to those reported for neonatal mortality in days 0–6 except very early NMRs on days 0–1 were higher than rates on days 2–6 in all sites but Guatemala (see Additional file 1: Tables S1 and S2).

## Discussion

In our study, we found that occurrence of all 8 CAD practices at delivery is associated with reduced risk of neonatal mortality in days 0–6 of life by 19%. In our Global Network sites, deliveries occurring in a hospital, birth attendants using a clean razor to cut the cord and new gloves during delivery, and appropriately initiating breastfeeding and skin-to-skin care, are associated with a decreased risk of early neonatal mortality. When all 8 intra- and post-partum CADs occurred, early neonatal mortality was lower than when all 5 intra-partum indicators and 0–2 post-partum indicators occurred, indicating the importance of early post-partum care of the newborn.

Several CAD indicators need closer examination as the results seem counterintuitive. CAD2: delivery by a physician is associated with an increased risk of early neonatal mortality (day 0–6). Physicians are more frequently based at referral hospitals and also more likely to attend to pregnant women with comorbidities or complications of labor and delivery. CAD3: fetal heart rate assessed prior to delivery is associated with a higher risk of day 0–6 neonatal mortality, possibly because fetal heart rate may more likely be monitored intra-partum when complications occur. CAD8: delayed bathing for more than 6 h is recommended as part of ENC thermal care, but this indicator was associated with increased day 0–6 neonatal mortality. However, there is again likely a bias as those infants who are unstable or seriously ill are also likely to receive delayed bathing. Neonates with delayed bathing were also more likely to be delivered by a physician and to not have initiated breastfeeding within 1 h of delivery (data not shown). These associations suggest that delayed bathing may not be an optimal indicator in a composite index of quality of intra-partum and post-partum care around delivery. Skilled Birth Attendant and Fetal monitoring are indicators of intra-partum care and most impactful in reducing rates of fresh stillbirth and neonatal mortality just after delivery. Therefore, they significantly increased the risk of 0–1 day mortality. Some of the sick neonates who survived beyond this period and were not well enough to be bathed died between day 2–6. So, delayed bathing was associated with deaths from day 2–6 and presence of skilled birth attendant and fetal monitoring ceased to remain significant for neonatal mortality beyond the first day after birth. CAD indicators that inform quality of care need to be reviewed to include indicators that assess training of providers in ENC and resuscitation and exclude those indicators around delivery whose occurrence may be influenced by the fetus/neonate being at risk of an adverse outcome. Additional post-partum candidate indicators that can be considered for a composite scoring system are immediate and thorough drying of baby, availability of functional bag and mask at the facility, delayed cord clamping and kangaroo mother care.

Our results indicating that ENC is associated with a reduction in early neonatal mortality are similar to others [20], but our composite index of the indicators has not been previously studied. The indices with the lowest coverage in our study, skin-to-skin contact between mother and baby, and early initiation of breastfeeding within the first hour of birth indicate significant room for improvement. We and others have shown an association of early initiation of breastfeeding within an hour of delivery and a reduction in neonatal mortality [21, 24]. Kangaroo mother care reduces neonatal mortality particularly for low birth weight babies [25], but remains difficult to achieve.

While presence of all 8 indicators as a composite index as a simple way to assess quality of care was associated with reduced early neonatal mortality, it is difficult to compare our results with the Utter Pradesh WHO Safe Birth Checklist Program because of the many differences in design and approach [14]. It is difficult to compare either study with the Rajasthan quasi-experimental study that also used the WHO Safe Birth Checklist due to differences in design and outcomes, as it has a quasi-experimental design and focus on stillbirths and very early neonatal death (within 3 days of birth) [15]. However, the variability in outcomes may provide clues to further simplifying the WHO checklist to a more limited set of indicators that have the most influence on neonatal mortality, as well as assessing other factors such as availability of equipment, drugs and supplies in different locations and may be of major importance for optimizing measurement of quality of care globally.

Our study has important strengths. First, our population-based registry cohort and database have excellent quality control. Second, our study population includes a diverse multi-regional rural population undergoing labor and delivery in a wide range of government and private health care facilities. Third, our composite index is evidence-based and easy to collect and monitor. Our study also has important limitations. First, availability of trained personnel to perform neonatal resuscitation was not evaluated, in part because of its complexity. Second, as described above, four of our CAD indicators are difficult to interpret because the indicator may be present due to the fetus/neonate being at risk of an adverse outcome, rather than indicating good quality of care. Third, since clean delivery kits were provided when requested to facilities where registry participants delivered their babies, our results may not be generalizable beyond this population. Fourth, the study was not designed to assess indicators for home deliveries because women globally are being encouraged to deliver in facilities. Fifth, we did not evaluate different weights of the indices in the composite index. Sixth, we do not have a standardized severity of illness score for neonates or 1- and 5-min Apgar scores to understand how the status of the newborn impacts the postnatal care around delivery indicators. However, on further analysis of our data, babies had delayed bathing (usually an indicator of better care around delivery) when there was one or more maternal complication or presence of a neonatal condition, and in babies with gestational age of < 34 weeks and birth weight less than 1500 g. It was difficult to interpret the neonatal outcome when other combinations of indicators (category iii as defined in the methods) were present, likely because of the heterogeneity of the more than 180 combinations of the 8 CAD indicators that were not included in category (i) or (ii) as defined above. Finally, since the GN facilitated access to clean delivery kits, CAD4 and CAD5: use of new gloves and use of a clean razor to cut the cord (part of ENC hygienic cord and skin care) were present in almost all deliveries. The effect of these two CADs requires further investigation in populations with less frequent use of ENC hygienic cord and skin care practices. Our study indicates that all clinically important practices of CADs may not have a similar impact on improving the outcomes of the babies. So developing composite indices of indicators is challenging. Further research is needed to select and prioritize indicators that help to improve the quality of care around delivery to improve neonatal outcomes.

## Conclusion

This study has important implications for future research and clinical practice, as reducing neonatal mortality has become an increased area of focus in the Sustainable Development Goals. Simple ways to improve and monitor quality of facility-based perinatal care are urgently needed, despite the complexity of the WHO’s recently developed framework and standards for health care facilities. Our simple composite index of quality of care is associated with reduced neonatal mortality, but could be refined further.

## Supplementary information

**Additional file 1: Table S1.** Day 0–1 neonatal mortality by received by care around delivery indicator. **Table S2.** Day 2–6 neonatal mortality by received care around delivery.

## Data Availability

The minimal dataset that outlines the findings of the provided results will be made available on request.

## Abbreviations

UN: United Nations
WHO: World Health Organization
ENC: Essential Newborn Care package
GN: Global Network
MNHR: Maternal Newborn Health Registry
CAD: Care around delivery
NICHD: Eunice Kennedy Shriver National Institute of Child Health and Human Development
RTI: Research Triangle Institute International
