# Supplementary material for: Evaluating the effect of care around labor and delivery practices on early neonatal mortality in the Global Network’s Maternal and Newborn Health Registry
Source: Reprod Health. 2020 Nov 30;17(Suppl 2):156. doi: 10.1186/s12978-020-01010-w (PMC7708898; doi:10.1186/s12978-020-01010-w)
Supplement: Supplementary file 1 — Additional file 1: Table S1. Day 0–1 neonatal mortality by received by care around delivery indicator. Table S2. Day 2–6 neonatal mortality by received care around delivery. [file 12978_2020_1010_MOESM1_ESM.docx]

**Additional Table S1. Day 0-1 neonatal mortality by received by care around delivery indicator.**

|  | Neonatal Mortality Day 0-1^1^ | | Risk of Neonatal Mortality Day 0-1 | | | |
| --- | --- | --- | --- | --- | --- | --- |
|  | Received Care Around Delivery | | Care vs. No Care, Unadjusted^2^ N=150,848 | | Care vs. No Care, Adjusted^3^ N=142,469 | |
| Care Around Delivery Indicators | Present n/N (Rate/1000) | Absent n/N (Rate/1000) | RR (95% CI) | P-Value | RR (95% CI) | P-Value |
| All 8 CAD indicators | 65/30,006 (2.2) | 1,779/120,842 (14.7) | 0.16 (0.09, 0.26) | <.0001 | 0.35 (0.27, 0.47) | <.0001 |
| CAD1: Delivery in a hospital vs. clinic | 1,200/91,437 (13.1) | 644/59,411 (10.8) | 0.87 (0.75, 1.01) | 0.06 | 0.93 (0.85, 1.03) | 0.18 |
| CAD2: Skilled birth attendant at delivery | 1,048/75,359 (13.9) | 796/75,489 (10.5) | 0.89 (0.68, 1.16) | 0.38 | 1.13 (1.00, 1.28) | 0.05 |
| CAD3: Fetal heart rate assessed prior to delivery | 1,607/142,177 (11.3) | 237/8,671 (27.3) | 0.90 (0.68, 1.19) | 0.44 | 1.12 (0.97, 1.30) | 0.13 |
| CAD4: New gloves for delivery | 1,830/150,463 (12.2) | 14/385 (36.4) | 0.78 (0.37, 1.66) | 0.52 | 0.83 (0.47, 1.48) | 0.54 |
| CAD5: Clean cord practices | 1,819/148,877 (12.2) | 25/1,971 (12.7) | 0.50 (0.29, 0.88) | 0.02 | 0.79 (0.46, 1.36) | 0.40 |
| CAD6: Early initiation of breast feeding (within 1 hour of birth) | 279/113,352 (2.5) | 1,565/37,496 (41.7) | 0.06 (0.04, 0.08) | <.0001 | 0.25 (0.20, 0.31) | <.0001 |
| CAD7: Skin-to-skin practices (immediately after birth) | 470/96,602 (4.9) | 1,374/54,246 (25.3) | 0.54 (0.41, 0.73) | <.0001 | 0.61 (0.52, 0.72) | <.0001 |
| CAD8: Delayed bathing (>6 hours of birth) | 1,791/135,742 (13.2) | 53/15,106 (3.5) | 3.80 (2.52, 5.71) | <.0001 | 1.79 (1.40, 2.27) | <.0001 |

^1^ Columns present n=the number of neonatal deaths on days 0-1; N=number of live births when CAD is present or absent; and the day 0-1 neonatal mortality rate per 1,000 births within each care around delivery indicator for care present or absent.

^2^ Unadjusted relative risks, 95% confidence intervals and p-values are obtained from a Poisson model for day 0/1 neonatal mortality including the 8 care around delivery indicators and site with generalized estimating equations to account for the correlation of outcomes within cluster. The relative risks and p-values for all 8 CAD indicators index come from a separate Poisson model including the all 8 CAD indicators index and site.

3 Adjusted relative risks, 95% confidence intervals and p-values are obtained from a Poisson model for early neonatal mortality including the 8 care around delivery indicators and site adjusting for parity (0, 1-2, 3+(ref)), delivery mode(vaginal, cesarean (ref)), at least one maternal condition (yes, no (ref)), gestational age < 34 weeks (yes, no (ref)), birth weight < 1500g (yes, no (ref)) and at least one neonatal condition (yes, no (ref)) with generalized estimating equations to account for the correlation of outcomes within cluster. The relative risks and p-values for all 8 CAD indicators index come from a separate Poisson model including the all 8 CAD indicators index, site and the covariates described above.

**Additional Table S2. Day 2-6 neonatal mortality by received care around delivery.**

|  | Neonatal Mortality Day 2-6^1^ | | Risk of Neonatal Mortality Day 2-6 | | | |
| --- | --- | --- | --- | --- | --- | --- |
|  | Received Care Around Delivery | | Care vs. No Care, Unadjusted^2^ N=149,004 | | Care vs. No Care, Adjusted^3^ N=140,848 | |
| Care Around Delivery Indicators | Yes n/N (Rate/1000) | No n/N (Rate/1000) | RR (95% CI) | P-Value | RR (95% CI) | P-Value |
| All 8 CAD indicators | 102/29,941 (3.4) | 979/119,063 (8.2) | 0.42 (0.31, 0.56) | <.0001 | 0.66 (0.53, 0.83) | 0.0004 |
| CAD1: Delivery in a hospital vs. clinic | 701/90,237 (7.8) | 380/58,767 (6.5) | 0.86 (0.71, 1.05) | 0.14 | 0.84 (0.71, 1.00) | 0.04 |
| CAD2: Skilled birth attendant at delivery | 619/74,311 (8.3) | 462/74,693 (6.2) | 0.96 (0.78, 1.17) | 0.66 | 1.10 (0.95, 1.28) | 0.19 |
| CAD3: Fetal heart rate assessed prior to delivery | 978/140,570 (7.0) | 103/8,434 (12.2) | 1.13 (0.88, 1.46) | 0.32 | 1.21 (0.90, 1.62) | 0.22 |
| CAD4: New gloves for delivery | 1,075/148,633 (7.2) | 6/371 (16.2) | 0.68 (0.32, 1.45) | 0.32 | 0.79 (0.32, 1.99) | 0.62 |
| CAD5: Clean cord practices | 1,062/147,058 (7.2) | 19/1,946 (9.8) | 0.83 (0.62, 1.12) | 0.21 | 1.12 (0.80, 1.59) | 0.50 |
| CAD6: Early initiation of breast feeding (within 1 hour of birth) | 412/113,073 (3.6) | 669/35,931 (18.6) | 0.16 (0.12, 0.22) | <.0001 | 0.49 (0.40, 0.60) | <.0001 |
| CAD7: Skin-to-skin practices (immediately after birth) | 479/96,132 (5.0) | 602/52,872 (11.4) | 0.95 (0.73, 1.22) | 0.67 | 0.96 (0.80, 1.17) | 0.71 |
| CAD8: Delayed bathing (>6 hours of birth) | 1,032/133,951 (7.7) | 49/15,053 (3.3) | 2.05 (1.52, 2.77) | <.0001 | 1.62 (1.21, 2.17) | <0.01 |

^1^ Columns present n=the number of neonatal deaths on days 2-6; N=number of live births when CAD is present or absent; and the day 2-6 neonatal mortality rate per 1,000 births within each care around delivery indicator for care present or absent.

^2^ Unadjusted relative risks, 95% confidence intervals and p-values are obtained from a Poisson model for day 2-6 neonatal mortality including the 8 care around delivery indicators and site with generalized estimating equations to account for the correlation of outcomes within cluster. The relative risks and p-values for all 8 CAD indicators index come from a separate Poisson model including the all 8 CAD indicators index and site.

3 Adjusted relative risks, 95% confidence intervals and p-values are obtained from a Poisson model for early neonatal mortality including the 8 care around delivery indicators and site adjusting for parity (0, 1-2, 3+(ref)), delivery mode(vaginal, cesarean (ref)), at least one maternal condition (yes, no (ref)), gestational age < 34 weeks (yes, no (ref)), birth weight < 1500g (yes, no (ref)) and at least one neonatal condition (yes, no (ref)) with generalized estimating equations to account for the correlation of outcomes within cluster. The relative risks and p-values for all 8 CAD indicators index come from a separate Poisson model including the all 8 CAD indicators index, site and the covariates described above.
